# Granulomatous arteritis limited to the skin: case report and etiologic differential diagnosis^☆^

**DOI:** 10.1016/j.abd.2020.10.021

**Published:** 2022-03-21

**Authors:** Diego Henrique Morais Silva, Neusa Yuriko Sakai Valente, Agatha Ramos Oppenheimer, Anna Karoline Gouveia de Oliveira

**Affiliations:** Department of Dermatology, Hospital do Servidor Público Estadual de São Paulo, São Paulo, SP, Brazil

Dear Editor,

Vasculitis constitutes a heterogeneous group of conditions characterized by inflammation in the blood vessel wall, with narrowing or occlusion of the vascular lumen. A 43-year-old man, without comorbidities, had presented multiple erythematous nodules on the anterior surface of the lower limbs for the past ten years, with outbreaks and remissions (Fig. 1). He had no local or systemic symptoms. A skin biopsy was performed and histopathology disclosed a nodular infiltrate with epithelioid histiocytes and multinucleated giant cells, on the wall and around a medium-caliber vessel, with occlusion of the lumen by a fibrinoid thrombus (Figure 2, Figure 3). Verhoeff-van Gieson staining demonstrated the presence of an internal elastic lamina in the wall of the affected vessel (Fig. 2B). Fungi and AFB screening using Grocott and Faraco stains, respectively, were negative. Chest and sinuses tomography showed no alterations, as well as whole blood count, renal function, and measurement of complement fractions. As for ANCA, it was positive for a-ANCA 1/20 (negative for c-ANCA and p-ANCA).

**Figure 1:**
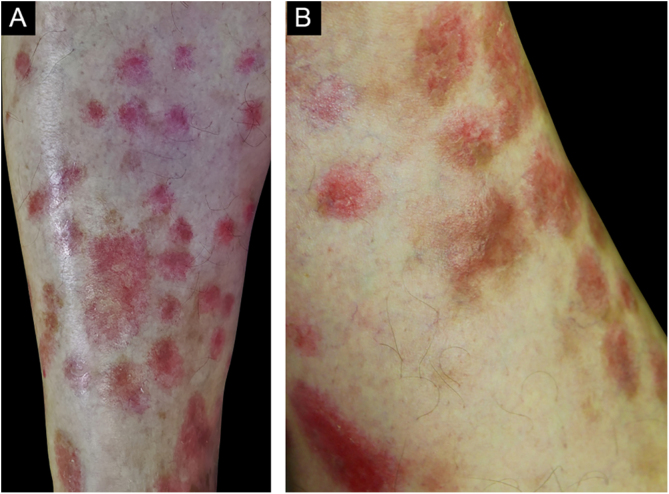
(A and B), Erythematous nodules on the extensor surface of the lower limbs.

**Figure 2:**
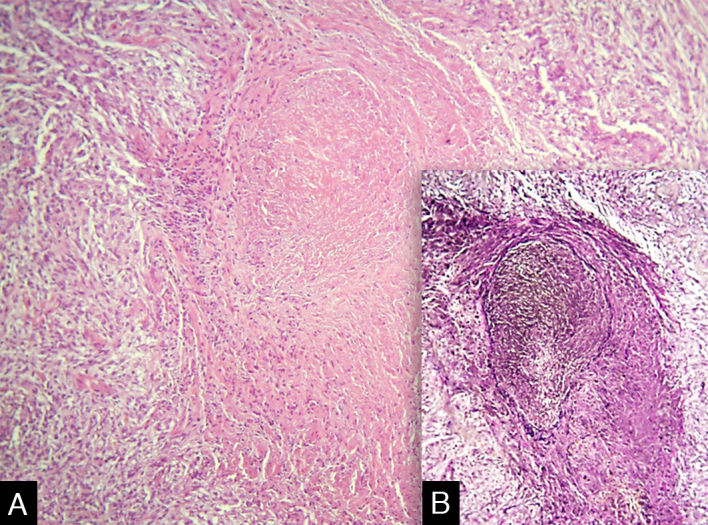
(A) Infiltrate in the wall of a medium-caliber vessel with the lumen occluded by a fibrinoid thrombus (Hematoxylin & eosin, ×200). (B) Presence of the internal elastic lamina (Verhoeff-van Gieson).

**Figure 3:**
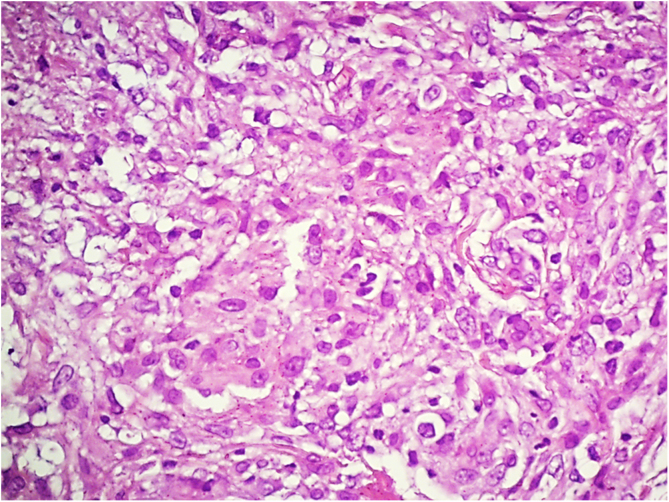
Detail of the infiltrate, consisting predominantly of epithelioid histiocytes (Hematoxylin & eosin, ×400).

Based on the histopathological findings, the diagnosis was granulomatous vasculitis. The term granulomatous vasculitis can be confusing, as it is used in two different contexts: (1) granulomatous inflammation in the vessel wall and (2) extravascular granulomatous inflammation associated with necrotizing vasculitis. The latter, better termed “vasculitis with granulomatosis”, has as prototypes granulomatosis with polyangiitis (GPA) and eosinophilic granulomatosis with polyangiitis (EGPA), which rarely have true granulomatous arteritis.^1^

Granulomatous vasculitis is a common finding in the skin lesions of large-vessel vasculitis.^1^ In temporal arteritis, there is usually a history of headache and muscle weakness, with the skin being unusually affected. Takayasu’s arteritis, which mainly affects the aorta and its branches, may also unusually affect the skin, with the histopathological finding of giant cell arteritis.^1^, ^2^ The patient described in the present case report had no symptoms consistent with these conditions.

Rarely, granulomatous arteritis is a cutaneous manifestation of systemic diseases such as sarcoidosis, inflammatory bowel disease, hepatitis C and post-herpes zoster. Moreover, there are cases associated with medications such as montelukast.^1^, ^3^ These possibilities were excluded through detailed anamnesis and complementary tests.

GPA and EGPA are ANCA-associated vasculitis, where histopathological analysis of the skin lesions usually discloses extravascular granulomas and small and medium - size vessels necrotizing vasculitis.^4^ More rarely, these vasculitis may present with granulomatous inflammation of the vessel wall and they are associated with systemic involvement, which was not seen in this patient.^1^

Atypical ANCA is characterized on indirect immunofluorescence by concomitant perinuclear and cytoplasmic staining, and it is mainly associated with drug-induced vasculitis.^4^ Although positive, it was not considered relevant to the clinical context, as, in addition to showing low titers, there was no history of medication or illicit drug use.

Cutaneous polyarteritis nodosa (PAN) manifests histopathologically as necrotizing arteritis.^2^ The literature has only one mention of the possibility of this condition manifesting granulomatous inflammation with multinucleated giant cells in the vascular lumen in 2 of 20 cases of PAN, which clinically had the presence of a nodule in 90%, livedo reticularis in 80%, and ulceration in 35% of the cases.^5^

This case report describes a case of granulomatous vasculitis limited to the skin. Despite extensive research, no causal factor was found, and clinical-laboratory follow-up as an outpatient was chosen.

## Financial support

None declared.

## Authors' contributions

Diego Henrique Morais Silva: Design and planning of the study; drafting and editing of the manuscript; collection, analysis, and interpretation of data; critical review of the literature.

Neusa Yuriko Sakai Valente: Approval of the final version of the manuscript; effective participation in research orientation; intellectual participation in the propaedeutic and/or therapeutic conduct of the studied cases; critical review of the manuscript.

Agatha Ramos Oppenheimer: Approval of the final version of the manuscript; critical review of the manuscript.

Anna Karoline Gouveia de Oliveira: Approval of the final version of the manuscript; critical review of the manuscript.

## Conflicts of interest

None declared.
